# SMAD3 deficiency promotes vessel wall remodeling, collagen fiber reorganization and leukocyte infiltration in an inflammatory abdominal aortic aneurysm mouse model

**DOI:** 10.1038/srep10180

**Published:** 2015-05-18

**Authors:** Xiaohua Dai, Jianbin Shen, Neeraja Priyanka Annam, Hong Jiang, Edi Levi, Charles M. Schworer, Gerard Tromp, Anandita Arora, Mary Higgins, Xiao-Fan Wang, Maozhou Yang, Hui J. Li, Kezhong Zhang, Helena Kuivaniemi, Li Li

**Affiliations:** 1 Department of Internal Medicine; 2 Center for Molecular Medicine and Genetics; 3 Cardiovascular Research Institute; 4 Department of Biochemistry, Wayne State University, Detroit, MI 48201; 5 Department of Pathology, Veterans Affairs Medical Center, Detroit, MI 48201; 6 The Sigfried and Janet Weis Center for Research, Geisinger Health System, Danville, PA 17822; 7 Department of Pharmacology and Cancer Biology, Duke University, Durham, NC 27710; 8 Bone and Joint Center, Henry Ford Hospital, Detroit, MI 48202; 9 Department of Medicine, University of Massachusetts, Worcester, MA 01655.

## Abstract

TGF-β signaling plays critical roles in the pathogenesis of aneurysms; however, it is still unclear whether its role is protective or destructive. In this study, we investigate the role of SMAD3 in the pathogenesis of calcium chloride (CaCl_2_)-induced abdominal aortic aneurysms (AAA) in *Smad3*^−/−^, *Smad3*^+/−^ and *Smad3*^*+/+*^ mice. We find that loss of SMAD3 drastically increases wall thickening of the abdominal aorta. Histological analyses show significant vessel wall remodeling with elastic fiber fragmentation. Remarkably, under polarized light, collagen fibers in the hyperplastic adventitia of *Smad3*^−/−^ mice show extensive reorganization accompanied by loosely packed thin and radial collagen fibers. The expressions of matrix metalloproteinases including MMP2, MMP9, and MMP12 and infiltration of macrophage/T cells are drastically enhanced in the vascular wall of *Smad3*^−/−^ mice. We also observe marked increase of NF-κB and ERK1/2 signaling as well as the expression of nuclear Smad2, Smad4 and TGF-β1 in the vessel wall of *Smad3*^−/−^ mice. In addition, we find that SMAD3 expression is reduced in the dedifferentiated medial smooth muscle-like cells of human AAA patients. These findings provide direct *in vivo* evidence to support the essential roles of SMAD3 in protecting vessel wall integrity and suppressing inflammation in the pathogenesis of AAAs.

Abdominal aortic aneurysm (AAA) is a common vascular disease among the elderly population in industrialized countries despite recent reduced prevalence and mortality due to decreased smoking rate, increased early detection and improved surgery^1^. AAA develops because of a combination of extensive vascular inflammation and maladaptive remodeling of the aortic wall^1^,^2^,^3^,^4^. Although various clinical and laboratory studies have provided insights into the pathogenesis of AAA^2^,^5^, the underlying molecular mechanisms remain elusive.

The role of TGF-β signaling activation in the pathogenesis of aortic aneurysms is ill-defined and controversial^6^,^7^,^8^.For example, inhibition of TGF-β signaling ameliorates Marfan syndrome (MFS)-associated aneurysms^9^; on the other hand, such inhibition of TGF-β signaling exacerbates angiotensin II infusion-induced AAAs^10^. Mutations in TGF-β receptors and SMAD3 (a key component of TGF-β canonical signal pathways) occur in patients with aneurysms associated with Loeys–Dietz syndrome (LDS), MFS, aneurysm-osteoarthritis syndrome (AOS) and familial thoracic aortic aneurysms and dissections (FTAAD)^11^,^12^,^13^: these discoveries point to the complexity of the role(s) of TGF-β signaling in the pathogenesis of aneurysms^6^,^8^. Nevertheless, such complexity, context-dependence, and apparent inconsistencies are not surprising in view of the multifaceted critical role that TGF-β signaling plays in cell migration, proliferation, and differentiation and in maintaining extracellular matrix (ECM) integrity^14^.

It is well-established that the TGF-β canonical signaling pathway is mediated through the phosphorylation and interaction of R-SMAD (SMAD2 and SMAD3) and co-SMAD (SMAD4) to regulate gene transcription in the nucleus^6^. Among the SMAD proteins, SMAD3 plays a central role in regulating collagen expression, ECM deposition and fibrotic responses^15^,^16^.

Since the roles of TGF-β signaling in AAA formation are uncertain^10^,^17^, we designed this study to seek *in vivo* evidence for the role of SMAD3 (known as a key intracellular mediator of the TGF-β signal pathway) in AAA formation. We analyzed experimental mouse AAAs using the well-established CaCl_2_-induced mouse AAA model^18^ in a *Smad3* knockout mouse line that was generated by deleting the *Smad3* translation initiation site^19^. Our results demonstrate that SMAD3 plays a protective role in the pathogenesis of the experimental AAAs. Specifically, we find that SMAD3 deletion induces the activation of ERK1/2 and NF-κB signal pathways, and increases the expression of its homologous SMAD2 and SMAD4. This result is consistent with two recent studies^20^,^21^ aimed at determining the roles of SMAD3 mutation in TAA and/or AAA of human AOS using a *Smad3* knockout mouse line that was generated by deletion of the C-terminal activation domain^22^. Taken together, these three independent studies using two different *Smad3* knockout mouse lines demonstrate that SMAD3 is critical to protect the vessel wall from aneurysm formation. Since there is a subgroup of human AAAs showing significant wall thickening and tremendous inflammatory cell infiltration, our CaCl_2_-treated *Smad3* knockout mice can be used as a new valuable pro-inflammatory AAA mouse model.

## Results

### Loss of SMAD3 promotes AAA formation with marked vessel wall remodeling in response to CaCl_2_ treatment

**Figure 1:**
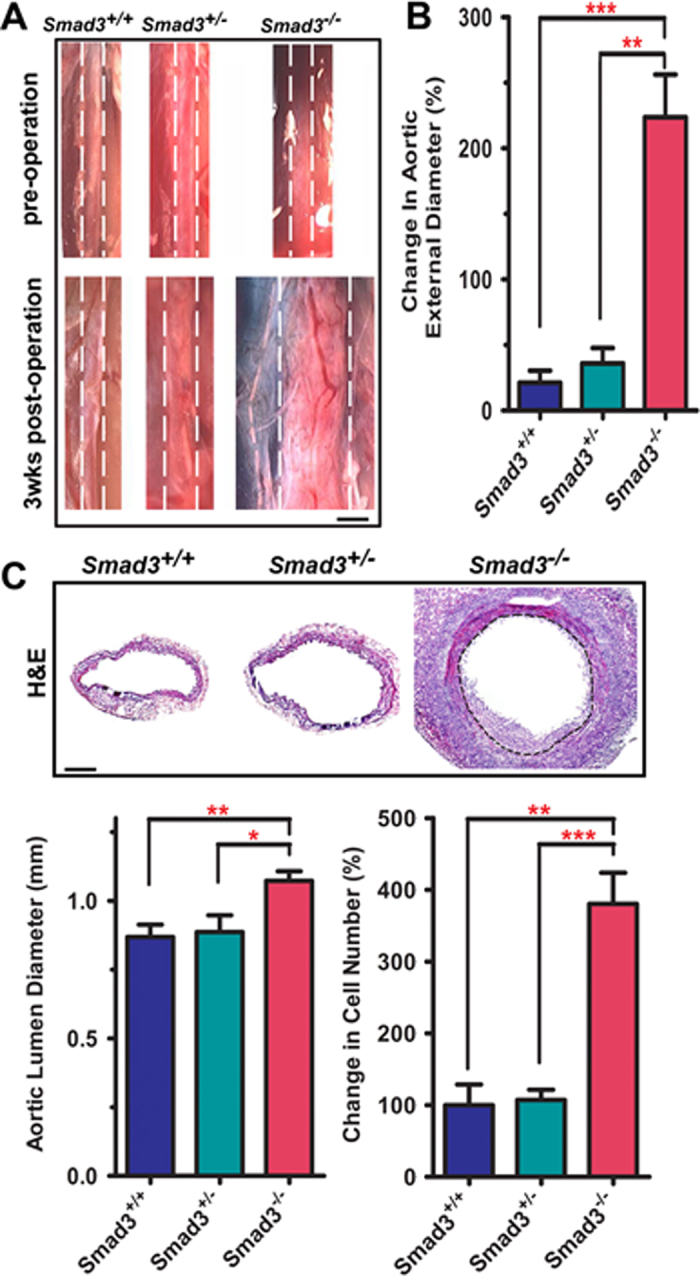
CaCl_2_ induces AAA formation in *Smad3*^−/−^ mice. **A**. Loss of Smad3 promoted AAA formation three weeks after CaCl_2_ perivascular application. White dashed lines were added to aid the identification of aortic anatomy. Scale bar: 1 mm. **B**. Change in size of the abdominal aorta among *Smad3*^+/+^, *Smad3*^*+/−*^, and *Smad3*^−/−^ mice three weeks after the CaCl_2_ treatment. Data are presented as the increased size of aortic external diameter (post-pre operation) over the size of its pre-operation (%). Values are mean ±SEM from *Smad3*^+/+^(n = 5), *Smad3*^*+/−*^(n = 4), and *Smad3*^−/−^ (n = 5) mice. **P < 0.01; ***P < 0.001. **C**. Representative images of H&E staining. Black dashed lines mark the internal elastin lamina of the aortic wall of *Smad3*^−/−^. The external elastin lamina was partially degraded. Scale bar: 100 μm. Bar graphs represent the aorta lumen diameter (left) and the total number of nuclei (right) in the whole vessel wall of the aorta from *Smad3*^−/−^ (n = 11) compared with *Smad3*^+/+^ (n = 5) and *Smad3*^*+/−*^ (n = 5). *P < 0.05; **P < 0.01; ***P < 0.001.

To explore the roles of SMAD3 in the pathogenesis of AAA, we analyzed the CaCl_2_-induced AAAs using the *Smad3* knockout mice that was generated by replacing the translation initiation site of *Smad3* gene with *neomycine* (Supplemental Fig. S1A)^19^. Western blot (WB) analyses confirm that the expression of SMAD3 in the resulting *Smad3*^−/−^ mice is abolished (Supplemental Fig. S1B). CaCl_2_ treatment induced drastic overall increase of the abdominal aorta in *Smad3*^−/−^ mice compared with the *Smad3*^*+/−*^ and *Smad3*^*+/+*^ control mice (Fig. 1A, Supplemental Fig. S1C). Before the treatment, the maximum aortic external diameters of *Smad3*^*+/+*^*, Smad3*^*+/−*^, and *Smad3*^−/−^ were of similar size at about 0.66 mm. Three weeks after the treatment, the maximum external aortic diameter in Smad3^−/−^ mice increased to about 2.0 mm and showed an obvious 223% increase when compared with its pretreatment size (Fig. 1B). The aortic sizes of *Smad3*^*+/−*^ and *Smad3*^*+/+*^ mice showed 36% and 21% increase respectively when compared with their size before the treatment (Fig. 1B). These results indicate that loss of SMAD3 promotes AAA formation in this experimental model.

Consistent with the increased aortic size, histopathological analyses revealed apparent changes in the vessel wall architecture of *Smad3*^−/−^ mice versus *Smad3*^*+/+*^ and *Smad3*^*+/−*^ mice: H&E staining showed that there is marked thickening of the wall with outward remodeling: this is distinct from most aneurysms showing thinning and degradation of vessel wall. We also observed the presence of neointima as well as enlarged media and adventitia in the vessel wall of *Smad3*^−/−^ mice (Fig. 1C). Despite the existence of neointima, the lumen diameter of the aorta in *Smad3*^−/−^ mice increased from 0.87 mm to 1.07 mm (a 24% increase) compared with *Smad3*^*+/+*^ mice (Fig. 1C, the left bar graph): this is consistent with the overall increase of the abdominal aorta. However, mice exhibited individual variation and not all sections from enlarged aorta had obvious neointima. In addition, the number of cells as counted by the number of nuclei in cross sections of the vessel wall of *Smad3*^−/−^ mice increased 380% compared with *Smad3*^*+/+*^ and *Smad3*^*+/−*^ control mice (Fig. 1C, the right bar graph).

To determine whether cell proliferation contributes to the increased cell number, we performed immunohistochemical (IHC) analyses using antibodies against Ki67, a nuclear cell proliferation marker. The result showed a marked increase in the number of proliferating cells indicated by Ki67 positive nuclei in *Smad3*^−/−^ mice compared with *Smad3*^*+/+*^ mice in response to CaCl_2_ treatment (Supplemental Fig. S2). This result suggests that cell proliferation contributes to the overall increase of the aortic size.

### *Smad3* ^−/−^ mice show marked elastin fragmentation and radical collagen fiber reorganization in the vessel wall of AAAs

**Figure 2:**
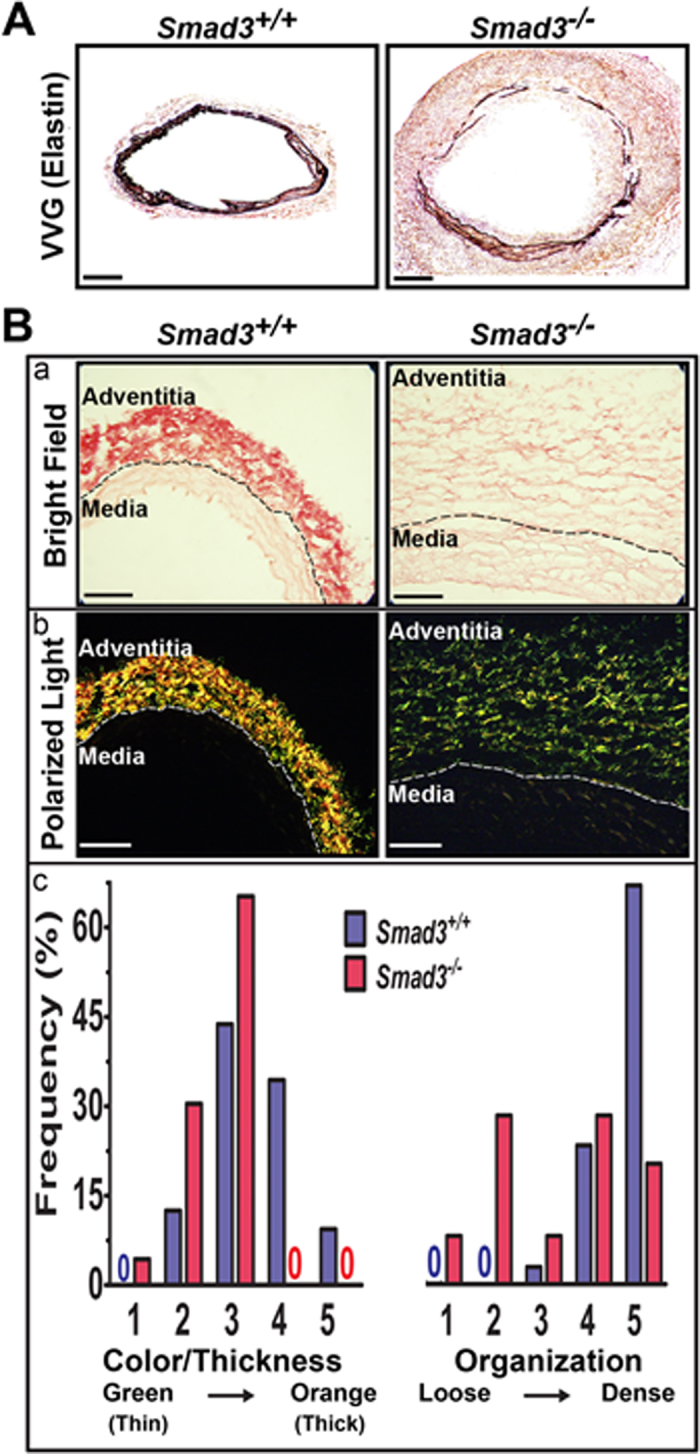
Elastin fragmentation and structural changes in collagen fibers in the aortic wall of aneurysms of *Smad3*^−/−^ mice. **A**. Representative images of Verhoeff-Van Gieson (VVG) elastin stained cross sections of abdominal aorta from *Smad3*^−/−^ mice (n = 7) and its *Smad3*^*+/+*^ control (n = 5). Scale bar: 100 μm. **B**. (a) Representative sections stained with Picrosirius red and viewed with bright field illumination. (b) Under polarized light, the tunica adventitia of *Smad3*^−/−^ mice contained more green (i.e., thin) collagen fibers than the *Smad3*^*+/+*^ mice. Also, the collagen in the adventitia of *Smad3*^−/−^ mice contained areas where the fibers were loosely packed. Scale bar: 70 μm. (c) Bar graphs show frequency distributions of histological scores for the color (thickness) and organization of collagen fibers. Refer to the on-line supplemental Method section for explanation of the scoring system. There were no green (thin) fibers or loosely packed fibers in *Smad3*^*+/+*^ mice, and there were no orange (thick) fibers in *Smad3*^−/−^ mice (as indicated by “0” in blue or red for Smad3^+/+^ or Smad3^−/−^ respectively). P = 0.0004 for the left graph; P < 0.0001 for the right graph (*Smad3*^*+/+*^ vs. *Smad3*^−/−^). *Smad3*^*+/+*^ (n = 7), *Smad3*^−/−^(n = 5).

To further characterize vessel wall remodeling in aneurysms, we analyzed elastin integrity by Verhoeff-Van Gieson (VVG) staining. Compared with the wild type control mice, elastin in the media of the vessel wall of *Smad3*^−/−^ mice exhibited extensive degradation three weeks after the surgery (Fig. 2A).

SMAD3 plays a central role in regulating collagen expression, ECM deposition and fibrotic responses^15^,^16^. Collagen fibers are the building blocks of the aortic wall, their thickness, organization and spatial distribution confer the mechanical properties (such as strength and stiffness) of the aortic wall^23^,^24^. We thus examined collagen expression and architecture in the vessel wall. Picrosirius red staining under bright light detected a decrease of collagen staining intensity in the tunica adventitia of the aneurysm in *Smad3*^−/−^ mice (Fig. 2Ba). Under polarized light, the architecture including the thickness and organization (orientation and spatial distribution) of collagen fibers was readily identified by the changes of colors and packing density respectively^25^,^26^ (Fig. 2B). As illustrated in panel 2Bb, the tunica adventitia of *Smad3*^−/−^ mice contained more green (i.e., thin) collagen fibers than the *Smad3*^+/+^ mice, Also, the collagen in the adventitia of *Smad3*^−/−^ mice tended to contain areas of disruption where the fibers were loosely packed. Such areas were seldom seen in *Smad3*^+/+^ mice. Although the predominant orientation of fibers in all of the sections was circumferential, fibers with a radial alignment were seen in central regions of the wall in some sections from *Smad3*^−/−^ mice. Thin and radial collagen fibers were only identified in the adventitia of aneurysms.

We used histology scores to quantify the color and organization of collagen fibers (see the Method section for explanation of the scoring system). The frequency distributions of the histological scores for collagen fiber thickness (indicated by color) and organization are shown in Fig. 2Bc. Overall, the color score was significantly lower for *Smad3*^−/−^ mice than for *Smad3*^+/+^ mice (P = 0.0004; Wilcoxon rank-sum test) as was the organization score (P < 0.0001; Wilcoxon rank-sum test). The median values for each distribution and their corresponding Inter Quartile Ranges (IQR) were as following: the median(IQR) values for color distribution were 3(4,3): 3(3,2) for *Smad3*^+/+^: *Smad3*^−/−^; the median(IQR) values of organization distribution were 5(5,4): 4(4,2) for *Smad3*^+/+^: *Smad*3^−/−^. Although the median values of color and organization are similar, the color and organization distribution are distinct (Fig. 2Bc). It should be noted that there were no green fibers and loosely packed fibers in *Smad3*^+/+^ mice, and there were no orange fibers in *Smad3*^−/−^ mice. Due to the small sample size (n = 3), we did not include data from *Smad3*^*+/−*^ mice; however, their median scores (color = 4, organization = 5) and distributions were similar to those of the wild type mice.

### Drastic increase in the expression and gelatinolytic activities of MMPs are detected in the abdominal aorta of *Smad3* ^−/−^ mice

**Figure 3:**
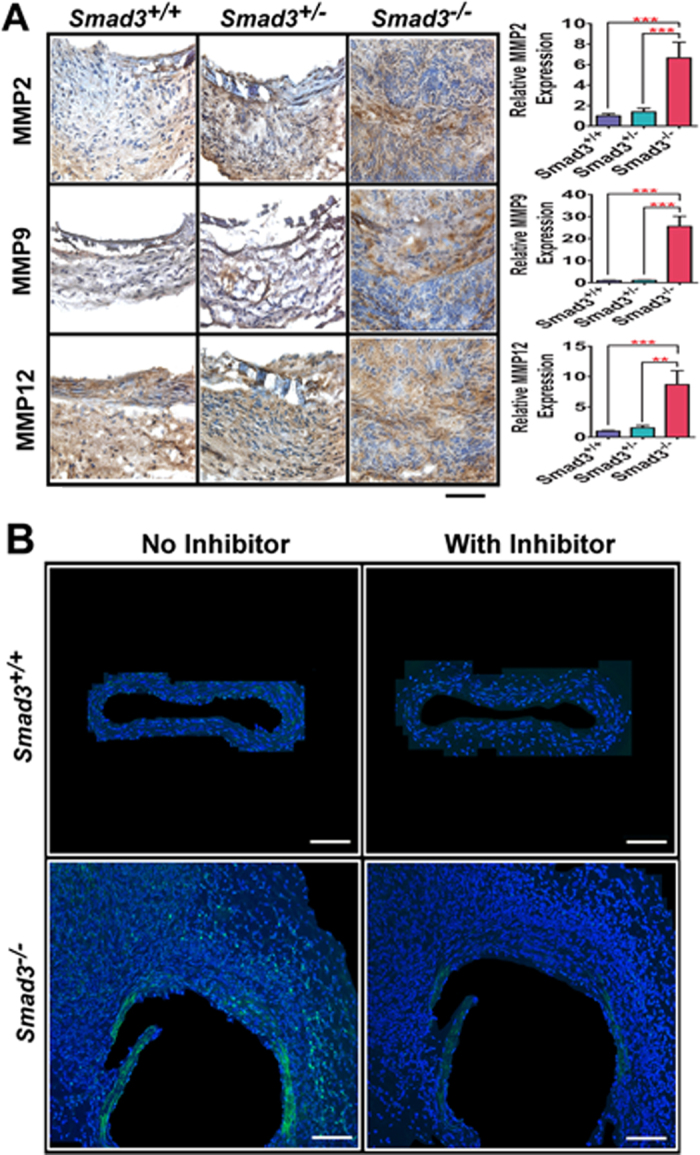
CaCl_2_treatment induces the expression of MMP2/MMP9/MMP12 and gelatinolytic activities of MMPs in the vessel wall in *Smad3*^−/−^ mice. **A**. Abdominal aortic sections from *Smad3*^*+/+*^, *Smad3*^*+/−*^ and *Smad3*^−/−^ mice treated with CaCl_2_ for three weeks were used for immunostaining with anti-MMP2, MMP9, and MMP12 antibodies. The relative antigen expression on the bar graphs indicates the fold changes of the antigen positive signal (shown in brown) over the total area of the aorta vessel wall from *Smad3*^−/−^ mice compared with their *Smad3*^*+/−*^ and *Smad3*^*+/+*^ control mice. *Smad3*^*+/+*^ (n = 6), *Smad3*^*+/−*^(n = 5) and *Smad3*^−/−^ (n = 5). **P < 0.01; ***P < 0.001. Scale bar: 40 μm. **B**. Representative images to show gelatinolytic activities of MMPs in abdominal aortic sections from *Smad3*^*+/+*^ and *Smad3*^−/−^ mice. Abdominal aorta from *Smad3*^−/−^ mice shows higher gelatinolytic MMP activities (the bright green florescence) than those from *Smad3*^*+/+*^ mice. Such MMP activities were diminished in the presence of the metalloenzyme inhibitor. *Smad3*^*+/+*^ (n = 5); *Smad3*^−/−^ (n = 4). Scale bar: 100 μm.

MMPs are responsible for the destruction of the orderly elastin and collagen network of the aorta^1^. MMP9, one of the prominently expressed MMPs in aortic aneurysms, works in concert with MMP2 to promote aneurysm progression^27^ MMP12 plays a direct role in elastin degradation in AAAs^28^. To determine whether MMPs contribute to the disruption of vessel wall integrity in *Smad3*^−/−^ mice, we performed IHC using antibodies against MMP2, MMP9 and MMP12. The expression of MMP2, MMP9 and MMP12 was detected in the aorta of all three Smad3 genotype mice after CaCl_2_ treatment; their expression was 6.6, 25.5 and 8.6-fold higher in *Smad3*^−/−^ mice for MMP2, MMP9 and MMP12, respectively, compared with the control mice (Fig. 3A).

To ensure that the increased expression of MMPs is accompanied by increased MMP gelatinolytic activities that accounts for the degradation of elastin and collagens, we performed *in situ* gelatinolytic zymography to detect MMP activities. In this assay, we used the OCT sections from abdominal aorta using highly quenched, fluorescein-labeled gelatin as a substrate that releases bright green fluorescence upon proteolytic digestion. Consistent with the increased expression of MMPs shown in Fig. 3A, abdominal aorta from *Smad3*^−/−^ mice show higher gelatinolytic MMP activities (Fig. 3B, the bright green fluorescence) than those from *Smad3*^*+/+*^ mice in both the media and the adventitia of the vessel wall. Such MMP activities are diminished in the presence of the metalloenzyme inhibitor 1,10- phenanthroline (Fig. 3B). This finding suggests that increased MMP activities contribute to vessel wall structure disruption observed in the aneurysms of *Smad3*^−/−^ mice.

### Leukocyte infiltration and NF-kB-ERK1/2 signaling are significantly increased in the abdominal aorta of *Smad3* ^−/−^ mice

**Figure 4:**
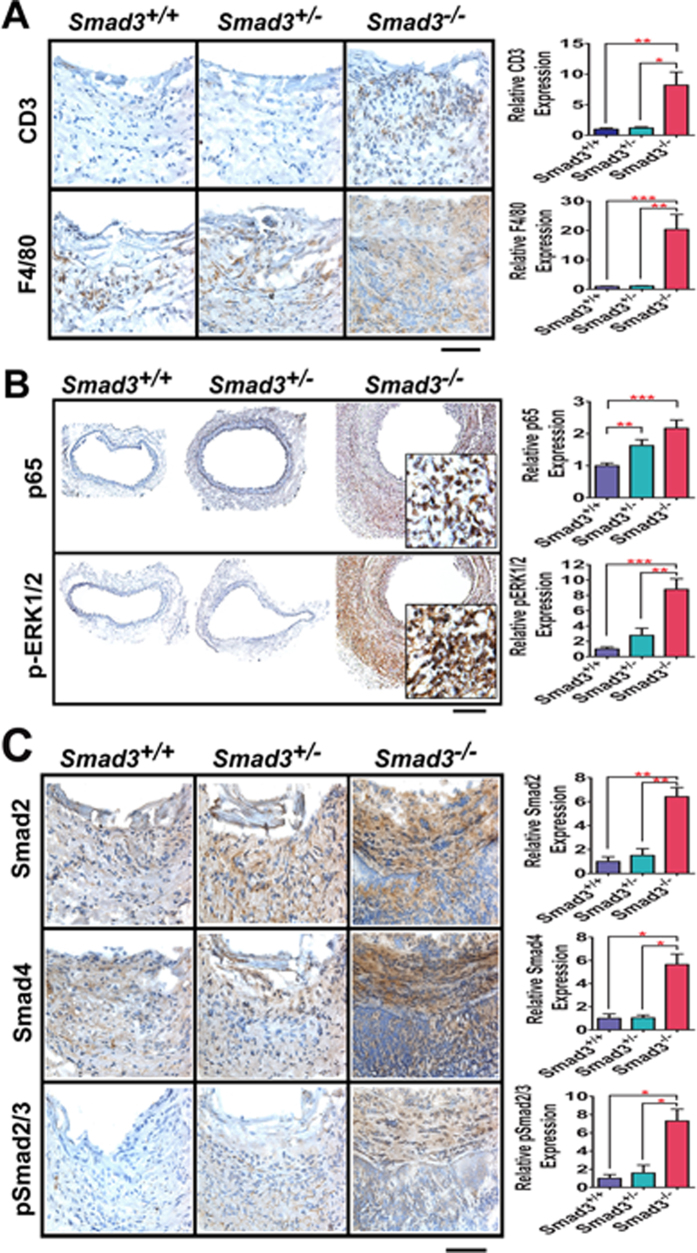
IHC assays detected robust leukocyte infiltration, activation of NF-ĸB and ERK1/2 signal pathways and upregulation of SMAD2 and SMAD4 in *Smad3*^−/−^ mice three weeks after CaCl_2_ treatment. **A**. Infiltration of T cells (CD3) and macrophages (F4/80) into the vessel wall from *Smad3*^−/−^ and their *Smad3*^*+/−*^ and *Smad3*^*+/+*^ control mice. Scale bar: 40 μm. **B.** The expression of nuclear p65 and pERK1/2 in the abdominal aorta using anti-p65 and anti-phosphorylated ERK1/2 antibodies. Scale bar: 100 μm. The insert shows the high magnification. **C**. The upregulation of Smad2 and Smad4 are detected in the vessel wall of *Smad3*^−/−^ and their *Smad3*^*+/−*^ and *Smad3*^*+/+*^ control mice. Scale bar: 40 μm. The relative antigen expression on the bar graphs indicates the fold changes of the antigen positive signal (shown in brown) over the total area of the aorta vessel wall from *Smad3*^−/−^ mice compared with their *Smad3*^*+/−*^ and *Smad3*^*+/+*^ control mice. Please note that there is no IHC signal in the lumen of the aorta because the aortas were perfused before being harvested. *Smad3*^+/+^ (n = 6), *Smad3*^*+/−*^(n = 5), and *Smad3*^−/−^(n = 5). *P < 0.05; **P < 0.01; ***P < 0.001.

Infiltrated leukocytes are a major source of MMPs production in the vessel wall^1^,^3^. IHC assays using antibodies against CD3 and F4/80 detected extensive infiltration of T lymphocytes and macrophages, respectively, throughout the aortic media and adventitia in *Smad3*^−/−^ mice (Fig. 4A). Compared with *Smad3*^*+/+*^ mice, the infiltration of T cells (CD3) and macrophages (F4/80) were increased 8.2- and 20.3-fold in *Smad3*^−/−^ mice (Fig. 4A). This observation demonstrates that loss of SMAD3 induces remarkable inflammatory responses in *Smad3*^−/−^ mice after CaCl_2_ treatment.

Since inflammation is prominent in the aneurysms, we examined the expression of p65 (aka RELA), the master regulator of cellular proinflammatory responses in the vessel wall of the aneurysms in *Smad3*^−/−^ mice (Fig. 4B). Please note that the p65 positive signals were localized in the nuclei. The expression of nuclear p65 showed 1.6-fold and 2.2-fold increase in *Smad3*^*+/−*^ and *Smad3*^−/−^ mice, respectively, compared with *Smad3*^*+/+*^ mice (Fig. 4B). Thus, Smad3 deficiency affects NF-kB expression level in a dosage-dependent manner.

ERK1/2 signal activation has been shown to play critical roles in the pathogenesis of aneurysm^29^,^30^. We, therefore, examined the activation of ERK1/2 signaling in the CaCl_2_-induced AAAs. IHC analyses revealed robust upregulation of phosphorylated ERK1/2 throughout the vessel wall of aneurysms in *Smad3*^−/−^ mice (Fig. 4B). The expression of phosphorylated ERK1/2 in *Smad3*^−/−^ mice was 2.8-fold and 8.8-fold higher than in *Smad3*^*+/−*^ and *Smad3*^*+/+*^ mice respectively.

### SMAD3 deficiency induces the upregulation of SMAD2, SMAD4 and TGF-β1

SMAD2 and SMAD4 share similar structure and overlapping function with SMAD36,14,15. We thus examined the expression of SMAD2 and SMAD4 by IHC assays. We found that the expression of SMAD2 and SMAD4 was upregulated in *Smad3*^−/−^ mice (Fig. 4C). The activation of SMAD2 in the nucleus as indicated by the phosphorylated SMAD2 also significantly increased in the aneurysms of *Smad3*^−/−^ mice using the antibody recognizing phosphorylated SMAD2/3 (Fig. 4C). *Smad3*^−/−^ mice do not express SMAD3 (Supplemental Fig. S1B). This finding suggests that the expression of both SMAD2 and SMAD4 is upregulated in *Smad3*^−/−^ mice.

SMAD2 and SMAD4 are the intracellular mediators of TGF-β1 signaling. The upregulation of SMAD2 and SMAD4 prompted us to examine the expression level of TGF-β1. Indeed, the expression of TGF-β1 is increased in the vessel wall of CaCl_2_ treated *Smad3*^−/−^ mice comparing with their wild type controls (Supplemental Fig. S3). Therefore, CaCl_2_ treatment induces the upregulation of TGF-β1 that in turn may contribute to the increased expression of SMAD2 and SMAD4 via an autocrine mechanism.

### SMAD3 expression is down-regulated in human AAAs

**Figure 5:**
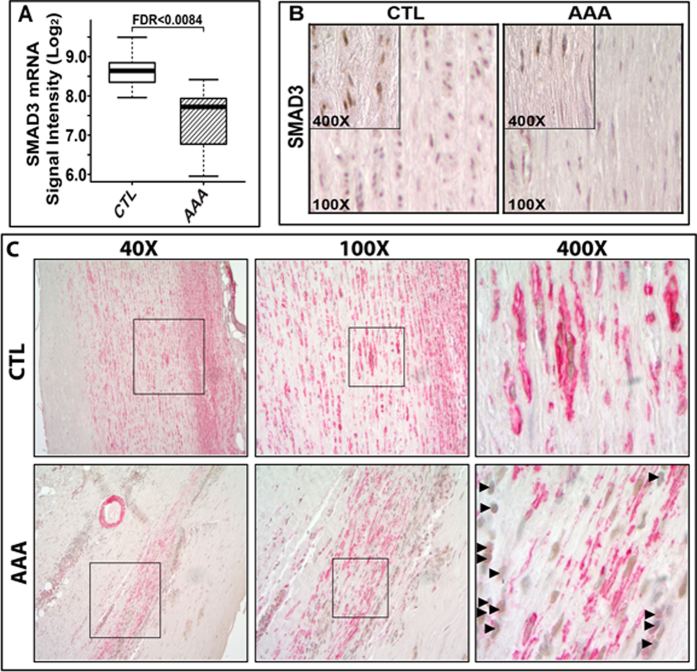
Reduced*SMAD3* mRNA levels and immunostaining of SMAD3 and ACTA2 in aortic wall samples from human AAA patients. **A**. Box and whisker plots of mRNA levels for AAA patients (n = 6) and infrarenal aortic samples from non-aneurysmal autopsy samples (n = 7) based on previous published microarray expression data^23^. RNA expression levels in signal intensity units are indicated on the ordinate-axis in logarithmic scale. Thick horizontal bars in the boxes indicate median values, boxes indicate interquartile range, and whiskers indicate range of non-outlier values. The difference between AAA and control (CTL) group was statistically significant after FDR correction. **B**. Immunostaining shows that SMAD3-positive nuclei (shown in brown) in the media of human AAA samples is lower than that in the non-aneurysmal infrarenal aortae (CTL). n=3. **C.** Medial layer of the vessel wall from AAA is highly degenerated. ACTA2-positive cells (shown in red) are limited to a few patches of spindle-shaped SMC-like cells. Few SMAD3-positive cells (shown in brown and indicated with arrowheads) are co-localized with ACTA2 positive cells in AAA samples. Co-localization of SMAD3 (shown in brown) and ACTA2 (shown in red) are observed in the medial layer of the vessel wall from control samples. Images (magnification at 40x, 100x and 400x) are shown.

To examine the clinical relevancy of SMAD3 deficiency in the pathogenesis of non-syndromic form of human AAA, we re-examined SMAD3 mRNA expression in our previously published microarray analyses of human aneurysmal and non-aneurysmal abdominal aorta tissue^31^: SMAD3 mRNA expression was significantly reduced in AAA tissue (Fig. 5A).

To access SMAD3 expression patterns in the aneurysms, we performed IHC assays using abdominal aorta tissues from both AAA patients and age–matched controls (Supplemental Table S). In the control aorta, the expression of SMAD3, predominantly in the nuclei rather than in the cytoplasm, was restricted to the medial smooth muscle cells (SMC) (Fig. 5B). The medial SMCs show high expression of ACTA2 (in red color) in non-aneurysmal autopsy control abdominal aorta (Fig. 5C).

As expected, the vessel wall from AAA tissues showed severe medial degeneration with extensive leukocyte infiltration and fibrosis (Fig. 5C, Supplemental Fig. S5): ACTA2 expression was high in vasa vasorum but low or absent in spindle shaped cells in the media of the vessel wall (Fig. 5C), a finding consistent with large AAA. Histological analyses showed high level of SMAD3 expression in inflammatory cells, ganglia cells and endothelial cells for neoangiogenesis throughout the vessel wall of AAA tissues where extensive fibrotic collagen and few medial SMCs were present (Supplemental Fig. S5).

In sections co-stained with ACTA2 and SMAD3 antibodies, SMAD3 IHC signals were less intense compared with ACTA2 IHC signals (Fig. 5C). We found that SMAD3 staining was mostly present in the infiltrated leukocytes in AAA samples (arrowheads in Fig. 5C). Consistent with the reduced SMAD3 expression in the preserved SMC-like cells in AAA sections (Fig. 5B), less SMAD3 staining was detected in AAA tissues in spindle shaped SMCs-like cells where ACTA2 expression was low or absent (Fig. 5C). Taken together, the data indicate that SMAD3 expression is reduced in medial SMC-like cells of the AAA tissues where ACTA2 is significantly downregulated, suggesting that SMAD3 deficiency may be associated with medial SMC degeneration.

## Discussion

**Figure 6:**
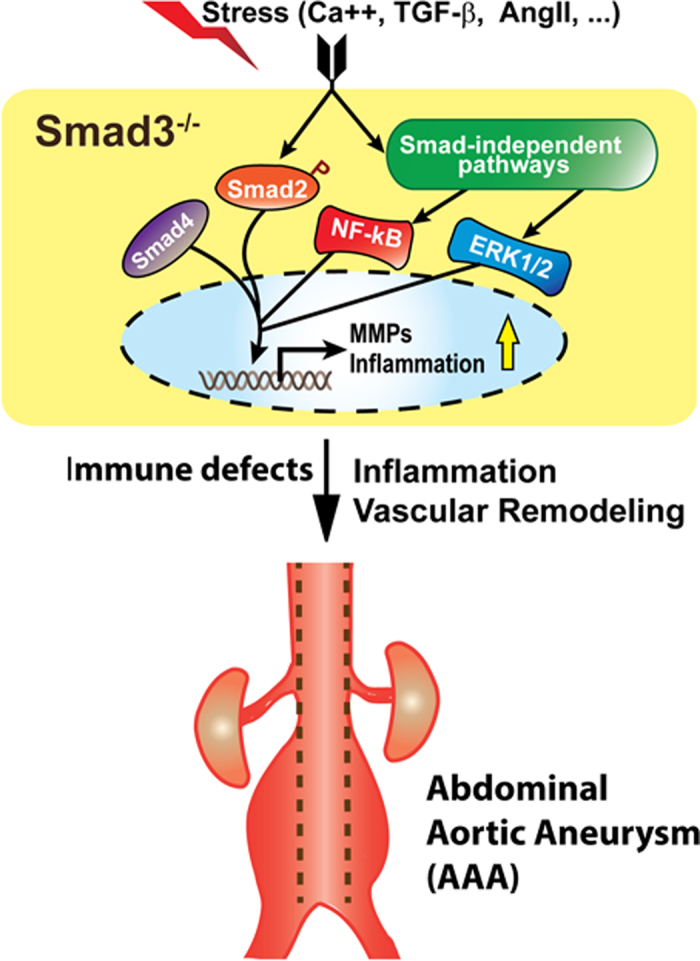
The schematic diagram of a hypothesized model showing that SMAD3 is required to protect vessel wall integrity and prevent aneurysm formation under stress conditions. In response to environmental stress (such as Ca^++^, TGF-β1 or AngII), SMAD-dependent canonical and SMAD-independent non-canonical signal pathways are activated in vascular cells including smooth muscle cells with defective SMAD3. The interplay of SMAD2, SMAD4, ERK1/2 and NF-κB signaling leads to increased MMP activities and the expression of proinflammatory genes, The resulting vascular inflammation, vessel wall remodeling (elastin degradation, collagen reorganization), and immune defects^19^,^22^ promote the formation of abdominal aortic aneurysm.

Our study provides strong *in vivo* evidence supporting the important roles of SMAD3 in protecting vessel wall integrity and suppressing inflammation in the pathogenesis of CaCl_2_-induced AAAs. These results are consistent with the essential roles of TGF-β signaling in inhibiting leukocyte infiltration and in promoting the synthesis of elastin and collagens to increase ECM deposition^32^. Interestingly, our present study using CaCl_2_-induced AAA mouse model is consistent with two recent studies^19^,^22^ using a different SMAD3 knockout mouse line. These three independent studies demonstrate that under stress condition disrupting SMAD3 expression activates SMAD-mediated TGF-β canonical pathway as well as SMAD-independent pathways including ERK1/2 and NF-kB signal pathways. The increased inflammation and the SMAD3 mutation-induced immune defects^19^,^22^ promote vessel wall remodeling and aortic aneurysm formation as shown in the proposed model (Fig. 6).

The differences between the two *Smad3* knockout mice should be pointed out: our *Smad3* knockout mice were generated by deleting the *Smad3* translation initiation site (i.e. SMAD3-null) (Supplemental Fig. S1A-B) and they do not show early sudden deaths; on the other hand, the mice used in the other two studies^20^,^21^ were generated by deletion of the C-terminal activation domain (i.e. SMAD3∆C) and typically die between 1-3 months. Since the SMAD3∆C lacks the TGFBR interaction domain and phosphorylation site for the SMAD3 activation, it is not surprising that loss of SMAD3 and the expression of the inactive SMAD3∆C protein induce aneurysm formation via similar mechanisms.

Regarding the more severe vascular phenotypes observed in SMAD3∆C mice^21^, the expression of the defective SMAD3∆C protein may contribute to the pathology. Given that human AOS is caused by SMAD3 mutation throughout the SMAD3 protein^12^,^13^, the SMAD3∆C knockout mouse line should be an excellent mouse model for human AOS. Interestingly, Hilhorst-Hofstee *et al* also reported a small interstitial deletion of chromosome 15, leading to the deletion of SMAD3 in a three-generation family with features of the aneurysms-osteoarthritis syndrome (AOS)^33^. Therefore, our SMAD3 null mouse line may also serve as an AOS mouse model. Although SMAD3 positive cells in AAA patients are likely to be proinflammatory cells by morphology (Fig. 5,5S), further study is needed to determine whether these cells are of dedifferentiated SMCs using a SMC lineage marker. Unfortunately, it is not easy to have access to a number of small AAAs to validate the protective roles of SMAD3 in human aneurysms.

It is interesting to note that SMAD2 activation is increased in aneurysms with SMAD3 deficiency in this study (Fig. 4C) and the report by Dr. Xia’s group^21^. The functional roles and regulatory mechanisms of SMAD2/3/4 in gene regulation are dependent on target genes and cell types^16^,^34^: for example, in mesenchymal progenitor cells, our previous study showed that SMAD3, not SMAD2, interacts with myocardin to modulate SMC gene transcription^35^. However, SMAD2 is now identified as the key regulator to control SMC gene transcription in neural crest derived VSMCs^36^. Interestingly, SMAD2 has been shown to be upregulated in thoracic aortic aneurysms that are of neural crest origins^37^. Indeed the underlying molecular mechanisms of the intricate interplay of TGF-β, SMAD2, SMAD3 and SMAD4 in the pathogenesis of aneurysm remains to be determined. Tissue-specific *Smad3* knockout studies should be used to address the functional roles of SMAD3 in each cell type in future studies.

The robust activation of ERK1/2 signaling in the present work is consistent with a series of recent studies in which blocking Smad-mediated canonical TGF-β signal transduction in different mouse aneurysm models invariably boosts the activation of the ERK1/2-mediated non-canonical TGF-β pathway^20^,^21^,^29^,^30^. The upregulation of TGF-β1 and ERK1/2 signaling may also account for the observed proliferation in the vessel wall^38^ (Fig. 1, Supplemental Fig. S3).These studies emphasize the complexity of TGF-β signaling pathways in the pathogenesis of aortic aneurysms. Further studies are needed to explore the molecular mechanisms of the complex yet delicate balance of SMADs, ERK1/2, and NF-kB signaling in the pathogenesis of aneurysms.

Since SMAD3 is the major intracellular mediator of the TGF-β1 signal pathway, the results of this study appear to support the protective roles of TGF-β1 in AAA described by Mallat’s group^10^. However, the underlying molecular mechanisms may be different: in our study, we observed the increase of TGF-β1 in the vessel wall of CaCl_2_-induced AAA in *Smad3*^−/−^ mice. When interpreting molecular mechanisms caused by gene mutation, one should take into account that genetic mutations induce a series of adaptive changes in the organisms; thus the functional role of a mutated gene in genetic diseases may be significantly different from the gene’s function under normal development. Different Smad3 mutations may elicit different alterations and adaptations in gene interaction network and signal pathway activation. Although three studies using two *Smad3* knockout mouse lines reveal the protective roles of SMAD3 via similar mechanisms, the functional roles of defective SMAD3 in human aneurysms appear to be more complex. For example, aneurysms from patients with SMAD3 mutations paradoxically display increased collagen expression along with increased SMAD2 phosphorylation and increased SMAD3 protein^13^. It is very likely that the upregulation of SMAD proteins and collagen is part of the adaptive response of SMAD3 mutations. The whole exome or whole genome sequence data in a large enough sample set may reveal potential association of rare variants in the *SMAD3* gene with AAA patients.

Finally, we would like to highlight that we are able to observe the unique architectural features of collagen fibers using the simple Picrosirius red staining combined with polarized light microscopy^25^,^26^ (Fig. 2B). This result is consistent with the study using electronic microscope observed by Dr. Tan’s group^20^. Aortic function and mechanical properties are influenced by the amount, organization, thickness, and density of collagen fibers^24^,^39^. Changes in collagen fiber architecture caused by molecular changes such as SMAD3 deficiency thus play important roles in the pathogenesis of AAA. Although collagen amount in AAAs are commonly examined by Picrosirius red staining, the important architectural properties of collagen fibers observable under polarized light are seldom accessed. Our finding of thin, disorganized fibers in *Smad3*^−/−^ mice is consistent with suggested roles of SMAD3 in structural integrity of the vessel wall. We believe that this histological approach could provide important insight into the pathophysiology of AAA.

In conclusion, this study reveals essential roles of SMAD3 in protecting the vessel wall integrity and suppressing inflammation. Our study suggests that the balance and interplay of NF-kB, ERK1/2, and SMAD2/4 signaling pathways have important roles in the pathogenesis of AAAs. Given the complexity of TGFβ signaling in aneurysm pathogenesis^6^,^32^, and aortic heterogeneity in embryonic origin and local hemodynamic stress^40^,^41^, caution should be taken when considering SMAD3 or TGF-β signaling pathways as the pharmacological target. Since there is a subgroup of human AAAs showing significant wall thickening and tremendous inflammatory cell infiltration, our CaCl_2_-treated *Smad3*^−/−^ mice can be used as a new valuable pro-inflammatory AAA mouse model.

## Methods

### Animal model

The animal protocols used in this study were approved by the Animal Investigation Committee at Wayne State University. The animal procedures performed conform the NIH guidelines (Guide for the care and use of laboratory animals). *Smad3*^*+/−*^ mice^19^ were maintained at C57BL6 genetic background. *Smad3*^*+/−*^ mice are fertile and phenotypically indistinguishable from the wild type mice.

### Aneurysm induction

AAAs were induced with CaCl_2_ using age matched male *Smad3*^−/−^*, Smad3*^*+/−*^ and *Smad3*^*+/+*^ at 8-12 weeks of age according to a well-established CaCl_2_-infusion protocol^42^,^43^. After anesthesia using 2% Avertin intraperitoneally (0.25 mg/kg body weight), the abdominal aorta between the renal artery and the iliac bifurcation was exposed by blunt dissection. The aorta was photographed using the Nikon SMZ1500 stereo microscope (Nikon Instruments Inc.). Next, a piece of gauze soaked with 0.5 M CaCl_2_ was applied uniformly along the abdominal aorta from the left renal artery to the bifurcation for 10 minutes. The aorta and the abdominal cavity were then rinsed with 0.85% sodium chloride three times before the incision was closed. The non-steroidal anti-inflammatory agent, carprofen (at 5 mg/kg in 0.5 ml of normal saline) was then administered to minimize the pain caused by the procedure. All mice were allowed to recover under a warm blanket and closely monitored until they regained consciousness. Mice were then placed into a clean cage and were kept under standard housing condition.

### Aorta collection and size measurement

Three weeks after surgery, the mice were anesthetized intraperitoneally using 2% Avertin (0.25 mg/kg). The abdominal aorta was then exposed for photography using the Nikon SMZ1500 stereo microscope. After cardiac perfusion using 4% paraformaldehyde, the abdominal aorta from the left renal branch to the bifurcation was removed, embedded in optimal cutting temperature (OCT) medium (Tissue-Tek® O.C.T. Compound, product code #4583) and sectioned at a thickness of 6 μm.

### Aorta size determination

The abdominal aorta filled with blood in live mice reflects aorta size more closely than aorta harvested postmortem. Digital images of the abdominal aorta both before and three weeks after CaCl_2_ treatment were thus taken and used to determine the external aortic diameter for each mouse. The diameter of the aorta is the average of three measurements of the maximum external aortic diameter. The diameter of the aorta at the end of CaCl_2_ treatment was expressed as a percentage increase from its respective initial diameter.

### Histology and immunohistochemical (IHC) analyses

The histology and IHC staining as described before^44^,^45^ were performed using OCT sections at comparable locations to the left renal artery along the abdominal aorta from *Smad3*^*+/+*^*, Smad3*^*+/−*^, or *Smad3*^−/−^ mice. Sections were stained with hemotoxylin-eosin (H&E), Verhoeff-Van Gieson (VVG; Elastin staining kit, Polysciences, Inc. Cat. #25089) or Picrosirius Red (Collagen Staining kit from Polysciences, Inc. Cat. #24901) following the manufacturer’s instructions. Digital images were obtained using Leica DM4000B light microscope (Leica Microsystems, Inc.). Digital images of H&E stained sections were used for morphometric analyses. The cell numbers in each section were counted by the nuclei using the Image-Pro Analyzer software (Media Cybernetics). The aortic lumen perimeter was determined by NIH Image J software (http://rsb.info.nih.gov/ij/)^46^.

For IHC analyses, the primary antibodies are the following: rabbit anti-Ki67 (a marker for cell proliferation, Abcam, ab15580), rabbit anti-MMP2 (Abcam, ab37150), rabbit anti-MMP9 (Abcam, ab38898), rabbit anti-MMP12 (Abcam, ab39876), rabbit anti-CD3 (Abcam, ab5690), rat anti-F4/80 (Abcam, ab6640), rabbit anti-p65 (aka RELA, one essential component of the NF-κB complex, Santa Cruz, sc-109), goat anti-SMAD2 (Santa Cruz, sc-6200), goat anti-pSMAD2/3 (Santa Cruz, sc-11769), goat anti-SMAD4 (Santa Cruz, sc-1909), and goat anti p-ERK1/2 (Santa Cruz, sc-16982). The primary antibodies were detected by the Avidin-Biotin Complex (ABC) staining systems (VECTASTAIN Elite ABC Kit, Vector Laboratories, Inc.). Sections were counterstained with Hematoxylin to visualize nuclei. Digital images of all mouse sections were obtained using the Leica DM4000B light microscope. Please note that there is no IHC signal in the lumen of the aorta because the aortas were perfused before being harvested. Threshold gated positive signal on IHC images was detected on the whole aorta vessel wall including the intima, media and adventitia layers. The antigen positive signals over the whole aorta cross section area was quantified using the integrative optical density (IOD) function in the Image-Pro Analyzer software (Media Cybernetics, Bethesda, USA).

### Collagen fiber structural organization assessment

Picrosirius red-stained sections were viewed with the bright field and polarized light. This combination allows structural properties of the collagen fibers to be assessed^26^,^47^. Specifically, the color changes as fiber thickness increases: from green to yellow to orange. Additionally, the optical properties of the birefringent fibers can be used to examine their orientation by rotating the section on the microscope stage and observing the angle at which the fibers become dark when viewed with linearly polarized light^25^.

The color and organization of collagen in the tunica adventitia of each section was graded on a 1 to 5 scale in a blinded fashion (by Dr. Peter Whittaker). For collagen fiber color, the scale was; 1 – predominantly green , 2 – mainly green with some orange and yellow, 3 – a mixture of all three colors, 4 – mainly orange and yellow with some green, 5 – predominantly orange. For collagen fiber organization, the scale was: 1 – extensive areas of disrupted and loosely packed around most of the circumference and abnormal structure (the presence of radially aligned fibers), 2 – large areas of collagen disruption (>50% of the vessel circumference), 3 – focal areas of collagen disruption (10–50% of the circumference), 4 – small areas of collagen disruption (<10% of the circumference), and 5 – densely packed collagen around the entire vascular circumference. The histological scores of collagen thickness (color) and structural organization (packing density) are given as medians with their inter quartile ranges (IQR) in the Result section and were compared using the Wilcoxon rank-sum test.

### *in situ* gelatinolytic zymograph

Gelatinolytic activity was demonstrated in OCT sections using DQ-gelatin as a substrate using EnzChek® Gelatinase/Collagenase Assay Kit following manufacturer’s instruction (Invitrogen, E12055). Cryostat sections of aorta were air-dried for 10 min. DQ-gelatin was dissolved in water at a concentration of 1 mg/ml. The stock was further diluted at 1:40 ratio using in 1x reaction buffer. About 200 μl of the diluted solution was put on top of the sections and covered with a coverslip. After 2-hour incubation at room temperature, slides were mounted with Vectashield with DAPI (Vectorlabs) and examined under the Leica DM4000B microscope. Specificity of the gelatinolytic activity due to MMP activities was determined with the presence of the metalloenzyme inhibitor 1,10- phenanthroline (1 mM). The inhibitor added at a concentration of 1 mM along with the gelatin substrate was able to reduce the gelatinolytic activity.

### Western blotting (WB) assay

Equal amount of protein lysates from the aorta of *Smad3*^+/+^, *Smad3*^*+/−*^, and *Smad3*^−/−^ mice were loaded on a 4-12% Bis-Tris NuPAGE Mini-gel (Invitrogen) for electrophoresis, followed by transfer onto an Immobilon-P membrane (Millipore). The membrane was blocked with 5% milk for 30 minutes, followed by primary antibody incubation overnight at 4 °C. After incubation with biotinylated secondary antibody for 30 minutes, the membrane was subject to enhanced chemiluminescence detection using SuperSignal West Pico Chemiluminescent Substrate (Pierce). The primary antibodies were anti-SMAD3 (1:1000, Abcam, ab14106), and anti-actin (1:5000 Cytoskeleton, Inc).

### Studies with human tissue samples

The research carried out was in accordance with the Declaration of Helsinki. The collection of the human tissues was approved by the Institutional Review Board of Wayne State University, Detroit, Michigan, USA and all subjects provided their informed and written consent to participate in this study. Aortic wall tissue specimens were collected from patients undergoing AAA repair operations at the Harper University Hospital, Detroit, Michigan. Non-aneurysmal infrarenal aortic samples were collected at autopsies^41^. Samples were stored in RNAlater for RNA isolations (Ambion), and in phosphate-buffered formalin (and embedded in paraffin) for histological and immunohistochemical analyses.

We used two microarray platforms to generate global mRNA expression profiles for both aneurysmal and non-aneurysmal human abdominal aorta. The details on these studies have been described previously^31^. The microarray data can be obtained at the Gene Expression Omnibus (GEO) database (Series# GSE7084; http://www.ncbi.nlm.nih.gov/geo/). A Box-and-Whiskers plot of *SMAD3* mRNA levels for AAA patients and infrarenal aorta tissues from non-aneurysmal autopsy based on microarray expression data (as described previously^31^) is shown in Fig. 5A.

Human aortic tissues from three AAA patients and age-matched non AAA patients (Supplementary Table S) were used for the IHC assay according to a protocol described previously^48^. Formalin-fixed paraffin sections (5-μm) were deparaffinized followed by antigen retrieval using microwave treatment in 0.01 M sodium citrate solution^13^ and incubated with SMAD3 primary antibody (Abcam ab28379, 1:100 dilution) or ACTA2 primary antibody (Abcam, ab5694) on an automatic immunostainer (Autostainer, DAKO, Carpinteria, CA). A biotinylated secondary antibody with avidin-biotin peroxidase amplification from DAKO was used and the signal detected using diaminobenzidine (DAB) as a chromogen. Images in the vessel wall from each sample were taken using an Olympus BX45TF microscope.

### Statistical analysis

Unless specified, all values in this study are expressed as mean±standard errors of the mean (SEM). Differences between two different groups were evaluated by unpaired Student *t* test using Prism software (Graphpad). Differences with p values <0.05 were considered statistically significant.

## Author Contributions

X.D., J.S., N.P.A., G.T., A.A., H.J.L. K.Z., H.K. and L.L. conceived and designed the experiments; X.D., J.S., N.P.A., H.J., E.L., C.M.S., G.T. and M.M. performed the experiments; X.D., J.S., N.P.A., E.L., C.M.S., G.T., A.A., M.M., analyzed the data; X.D., J.S., N.P.A., H.J., E.L., C.M.S., G.T., X.F.W., M.Y., H.J.L., K.Z.,and H.K. contributed reagents/materials/analysis tools; X.D., J.S., H.K. and L.L. wrote the manuscript. Overall responsibility of L.L.

## Additional Information

**How to cite this article**: Dai, X. *et al*. SMAD3 deficiency promotes vessel wall remodeling, collagen fiber reorganization and leukocyte infiltration in an inflammatory abdominal aortic aneurysm mouse model. *Sci. Rep.* **5**, 10180; doi: 10.1038/srep10180 (2015).

## Supplementary Material

Supporting Information
