# Supplementary material for: SMAD3 deficiency promotes vessel wall remodeling, collagen fiber reorganization and leukocyte infiltration in an inflammatory abdominal aortic aneurysm mouse model
Source: Sci Rep. 2015 May 18;5:10180. doi: 10.1038/srep10180 (PMC4434993; doi:10.1038/srep10180)
Supplement: Supporting Information [file srep10180-s1.pdf]

SMAD3 deficiency promotes vessel wall remodeling, collagen fiber reorganization and leukocyte infiltration in an inflammatory abdominal aortic aneurysm mouse model

Xiaohua Dai, Jianbin Shen, Neeraja Priyanka Annam, Hong Jiang, Edi Levi, Charles M. Schworer, Gerard Tromp, Anandita Arora, Mary Higgins, Xiao-Fan Wang, Maozhou Yang, Hui J. Li, Kezhong Zhang, Helena Kuivaniemi & Li Li

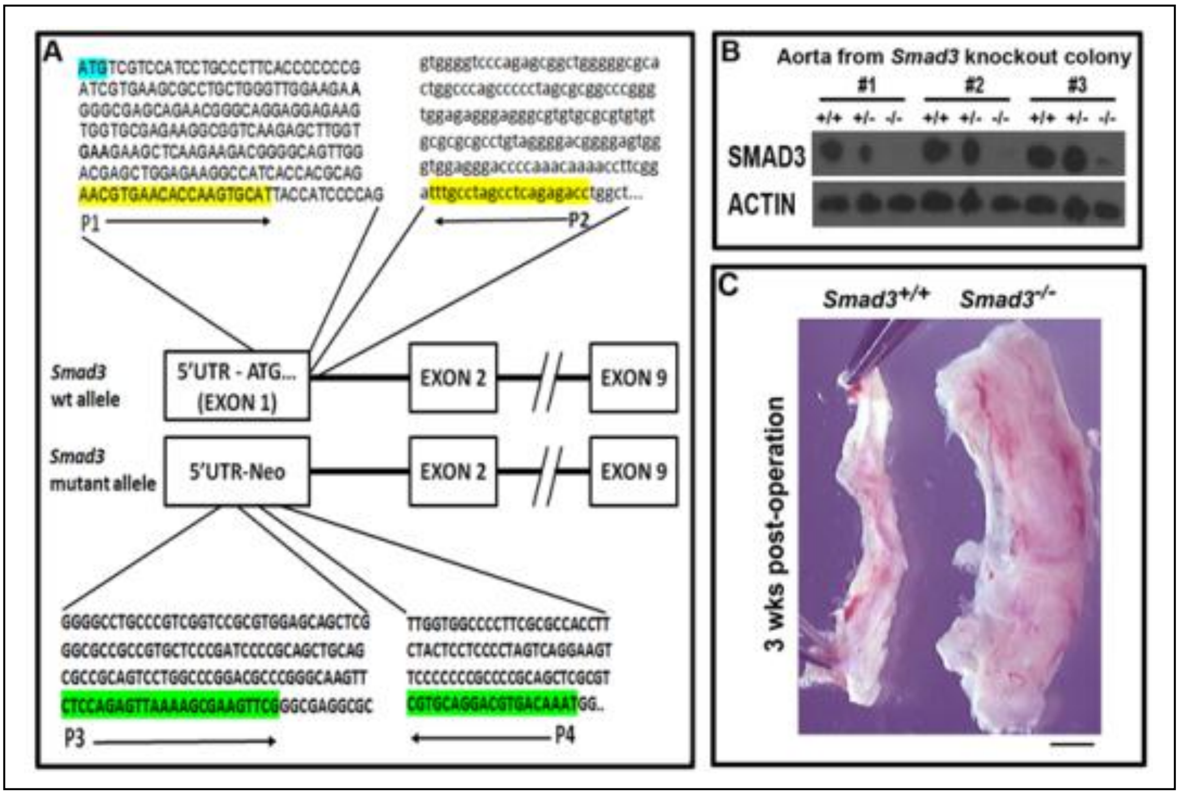

Figure S1 | (A) Sequence analyses confirm the replacement of the translational initiation site in the *Smad3* wild type (wt) allele by the *neomycin* gene in the *Smad3* mutant allele in the *Smad3* knockout mouse line[1]. ATG in light blue is the SMAD3 translation initiation site. P1 and P2 primer sequence (in bright yellow) for the *Smad3* wt allele; P3 and P4 primer sequence (in bright green) for the *Smad3* mutant allele.

Smad3$^{+/+}$, *Smad3*$^{+/-}$ and *Smad3*$^{-/-}$ mice were identified by genomic DNA PCR genotyping using primers specific for the wild type (WT) allele (P1: 5'-GGTCTCTGAGGCTAGGCAAA-3' and P2: 5'- AACGTGAACACCAAGTGCAT-3') and the mutated *Smad3* allele (P3: 5'- CTCCAGAGTTAAAAGCGAAGTTCG-3' and P4: 5'- ATTTGTCACGTCCTGCACGACG -3'): The PCR fragments are 190 bp and 350 bp respectively. The sequences of the PCR fragments confirm the replacement of the translational initiation site by the *neomycin* gene in the *Smad3*$^{-/-}$ mouse line.

(B) The western blot assay using the aorta protein lysate from the *Smad3* knockout mouse colony confirms that the expression of SMAD3 protein is abolished in *Smad3*$^{-/-}$ mice comparing with *Smad3*$^{+/+}$ and *Smad3*$^{+/-}$ mice. (C) Three weeks after $CaCl_2$ treatment, the abdominal aorta from *Smad3*$^{-/-}$ mice is significantly larger than the coresponding segment from *Smad3*$^{+/+}$ mice. Bar: 1mm.

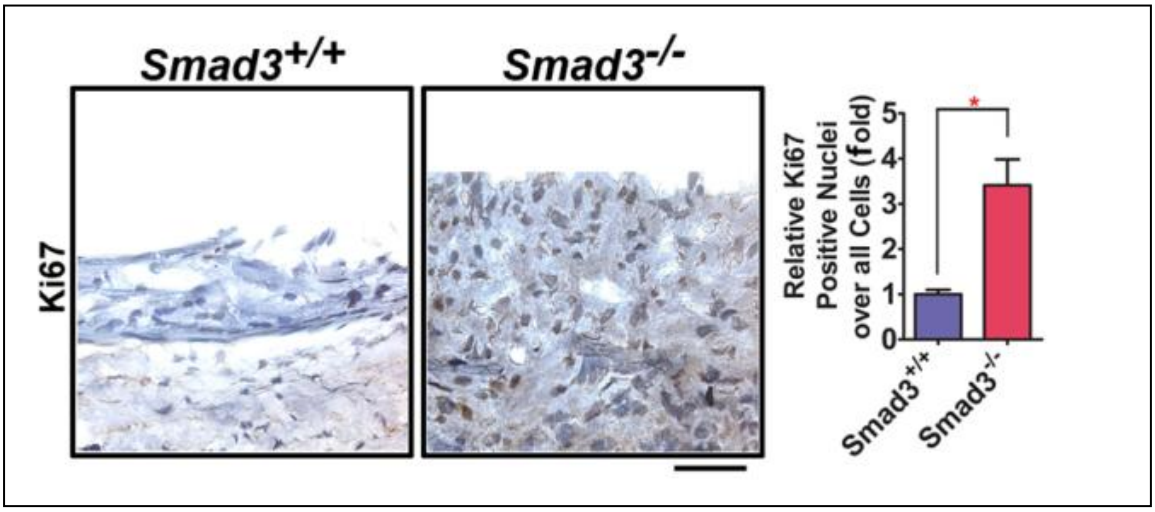

Figure S2 | $CaCl_2$ treatment induces cell proliferation in the vessel wall of *Smad3$^{-/-}$* mice. Abdominal aortic sections from *Smad3$^{+/+}$* and *Smad3$^{-/-}$* mice three weeks after $CaCl_2$ treatment were used for immunostaining using anti-Ki67 antibodies. Sections were counterstained with hematoxylin to visualize nuclei. The bar graph represents the comparison of the percentage of Ki67 positive nuclei (shown in brown) over all nuclei in the aorta vessel wall from *Smad3$^{-/-}$* mice and their *Smad3$^{+/+}$* control mice. n=5. Error bars represent the SEM. $^*P<0.05$. Scale bar: 40µm.

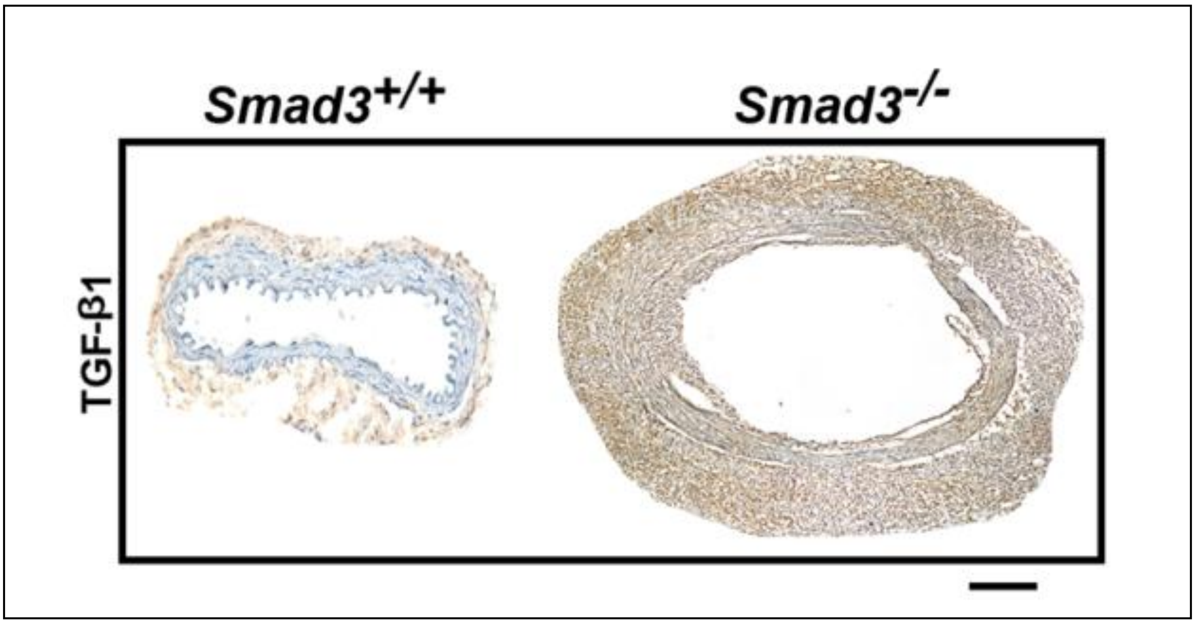

Figure S3 | TGF-β1 expression is increased in *Smad3$^{-/-}$* mice three weeks after $CaCl_2$ treatment. n=5. Scale bar: 100 µm.

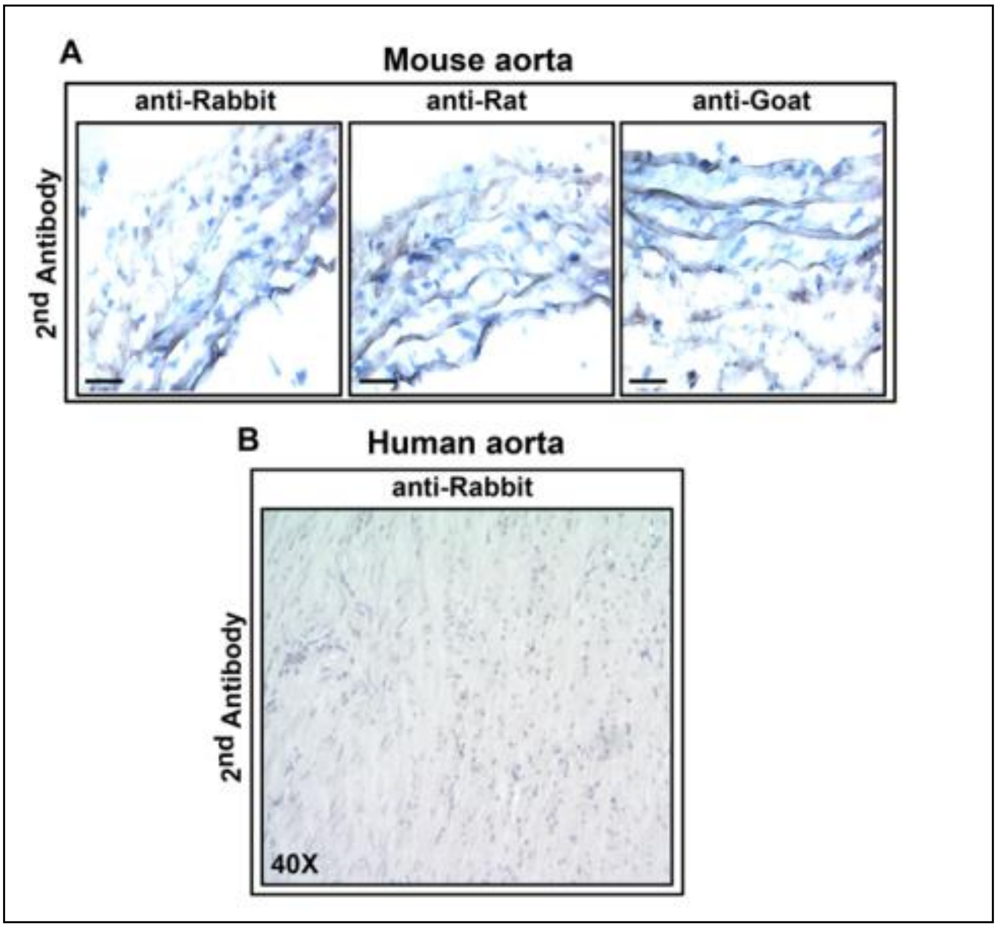

Figure S4 | Negative controls for IHC assays using 2nd antibodies corresponding to the species of primary antibodies do not show positive immunostaining signals (brown or red) in mouse aorta section (A, scale bar: 40μm) and human aorta section (B, magnification: 40X).

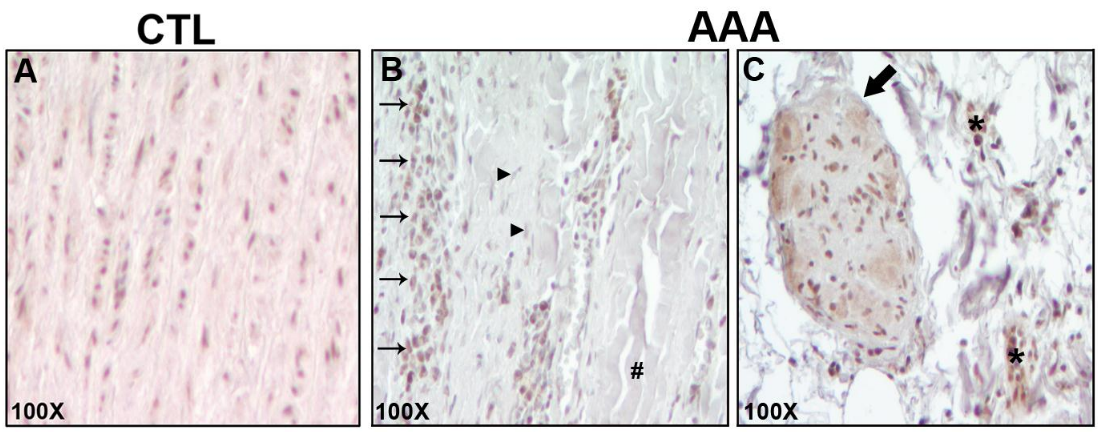

Figure S5 | SMAD3 expression in the infrarenal abdominal aortic tissues from AAA patients and non-aneurysmal autopsy controls (CTL) by IHC assays. (A) The expression of SMAD3 is restricted to the medial smooth muscle-like cells in the non-aneurysmal abdominal aorta control (CTL) tissues. (B) In the media of AAA tissues, SMAD3 is highly expressed in infiltrated leukocytes (thin arrows) by morphology, but low or absent in the smooth muscle-like cells (arrowheads). # indicates the wavy collagens (fibrosis). (C) In the adventitia of AAA tissues, SMAD3 is highly expressed in the ganglions (thick arrows) and endothelial cells (*) in neoangiogenesis. Magnification: 100X.

Table S | Human abdominal aortic tissues used for SMAD3 IHC staining. Infrarenal abdominal aorta tissues were obtained at autopsy (Control) or at open surgical aneurysm repairs (AAA). n=3. All donors and patients were Caucasian. M: Male; F: Female

Case ID	Age (yrs)	Sex	Cause of Death	Classification
ME0205	78	M	Cardiac Arrest	Control
ME0501	69	F	Fall (Head Trauma)	Control
ME0503	54	M	Cardiac Arrest	Control
WSU075	67	M		AAA
WSU080	64	F		AAA
WSU081	69	M		AAA
